# Economy in Baked Meats

**Published:** 1912-05-18

**Authors:** 


### INSTITUTIONAL HOUSEKEEPING.

### Economy in Baked Meats.

No form of cooking meat produces more palatable results than oven roasting when properly carried out. Unless care is taken, however, over each step of the process there is danger of waste; and in institutions it is not uncommon for the entire staff to go hungry because the precautions which every cook, however plain, ought to understand were not observed. The three principal dangers of waste in roasting lie in the choice of the meat, in the losses it undergoes during the roasting process, and in the management of the oven.

Roast meat can never form so cheap a meal as meat boiled or stewed, because it is only the choice parts of the animal which can give good results when roasted. Coarse, tough meat, or joints containing much gristle must be cooked at a moderate temperature in moist heat, or the tissues and gristle will become dry and hard instead of becoming soft, glutinous, and easy of digestion. Since in many institutions the matron has to make the best of supplies she does not herself order, she should firmly decline to allow unsuitable joints to be cooked in the oven. It is far better for the staff to grumble at monotony than for them to leave their rations hard and leathery on their plates. If housekeepers were more discriminating and alert in giving their orders to the cook, attention would be attracted to the quality of the meat, and contractors might be brought to account.

Meat loses more in weight when roasted or baked than when stewed. The average loss works out as follows:

| | |
|---|---|
| Roasted | About 30 per cent. |
| Baked | „ 25 per cent. |
| Stewed | „ 17–20 per cent. |

The loss arises mainly, however, through the melting out of fat, which remains in the tin as dripping, and through the evaporation of water. It is the concentration of flavour thus produced which renders roast meat appetising. But unless care is taken, much of the extractives which escape as gravy into the baking tin may be lost.

The main object in roasting is to conserve the juices and natural flavours of the meat by applying a very high temperature at the outset, to effect a sealing of the surface as in boiling. A thin outer crust of semi-carbonised albumen should be formed by this means, which will prevent excessive evaporation. A well-roasted joint or well-grilled chop may be recognised by its puffed-out appearance caused by the expansion of the fluids within this coating. When the meat has collapsed and looks shrivelled it is a sign that the oven was not hot enough to start with. After the preliminary sealing, the degree to which the heat is decreased must depend on the size of the joint; the larger the mass of meat the slower must be the cooking, otherwise the surface will be charred before the interior is cooked through. This is how waste occurs with careless cooks, who either dry up the small joints with slow cooking, or ruin the large joints by continuing to char the surface with intense heat which leaves them raw within.

Nothing can prevent undue desiccation of the meat while being baked but continual basting. A double baking tin should be used with a trivet to fit the inner one. The trivet raises the joint and prevents it from becoming sodden. The outer pan must be filled with water to obviate the unpleasant flavour imparted by burnt dripping. The flavour of the meat, however carefully cooked in other respects, will be unfavourably affected unless the oven is frequently ventilated by being opened for basting purposes. The oven must be free from any trace of burnt fat, and if there has been previous neglect, the shelves must be scraped with an old knife; the oven should be thoroughly scrubbed out with hot soda-water and a stiff brush twice a week.

By observing these elementary precautions, too often neglected by hurried institution cooks, roast meat, even though merely baked in an oven, will be not only a dish for an epicure, but also one of the most nourishing and easily digested items on the bill of fare.

### Housekeeping Queries.

How to Cleanse Wash-leathers.

L. M. G.—*The wash-leathers supplied to us for various purposes are becoming very dirty. The last set I ordered washed, but the result was they became hard, rough, and quite useless. Can this be prevented or is it inevitable? I should be glad to know.*

The method of washing was wrong, the rinsing and drying being faulty. Shake the leathers well to remove dust; make a strong soapy lather in warm, not hot, water, after adding to the latter one teaspoonful of ammonia to three pints of water. Knead and squeeze the leathers in this, but avoid rubbing, or rubbing soap on the leather, as this causes holes. Next rinse in two more soapy waters—never in clear water, the soap keeps the leathers soft. Squeeze dry without wringing and dry slowly, gently rubbing and pulling the leathers during the process to keep them soft and pliable.

Recipes for Using Cheese.

A Devonshire Reader.—*Our staff are very fond of cheese cooked in various ways. We have omelets, toasted cheese, Welsh-rarebit, and cheese pastry. Could you suggest other recipes to make greater variety? They must be simple and suitable for supper.*

Try *Cheese Pudding*, it is very simple, and far more digestible than toasted cheese. Mix together one teacupful of fresh white crumbs and the same of grated cheese. Well beat two eggs, add to them 1 pint of milk. Add these to the cheese and crumbs, season well and pour into a buttered pie-dish. Bake slowly until lightly browned and set. Serve at once. Another good dish is *Savoury Rice*. Boil 4 oz. of rice as for a curry. Whilst hot mix with it 3 oz. of grated cheese, 1 oz. of butter, and seasoning to taste. Stir over the fire until the cheese is melted and the whole very hot, then pile it up on a hot dish, with sippets of toast or fried bread round.

To Our Readers.

In this column questions are dealt with relating to cookery and household recipes, the duties of the domestic staff, estimates of quantities required for given numbers, and the cost of diet for patients and staff. Arrangements have been made to deal with samples submitted for examination and report on them.

